# Evaluation of country infrastructure as an indirect measure of dog-mediated human rabies deaths

**DOI:** 10.3389/fvets.2023.1147543

**Published:** 2023-05-09

**Authors:** Sarah C. Bonaparte, Janae Moodie, Eduardo A. Undurraga, Ryan M. Wallace

**Affiliations:** ^1^Poxvirus and Rabies Branch, Division of High Consequence Pathogens and Pathology, National Center for Emerging and Zoonotic Infectious Diseases, Centers for Disease Control and Prevention, Atlanta, GA, United States; ^2^New York University College of Global Public Health, New York, NY, United States; ^3^James A. Ferguson Emerging Infectious Diseases RISE Fellow, Centers for Disease Control and Prevention, Atlanta, GA, United States; ^4^Escuela de Gobierno, Pontificia Universidad Católica de Chile, Santiago, Chile; ^5^Millennium Initiative for Collaborative Research in Bacterial Resistance (MICROB-R), Santiago, Chile; ^6^Research Center for Integrated Disaster Risk Management (CIGIDEN), Santiago, Chile

**Keywords:** rabies—epidemiology, evaluation, country infrastructure, human rabies deaths, rabies surveillance, dog-mediated human rabies elimination, dog-mediated rabies, rabies

## Abstract

**Background:**

Rabies is a neglected disease, primarily due to poor detection stemming from limited surveillance and diagnostic capabilities in most countries. As a result, there is limited ability to monitor and evaluate country, regional, and global progress towards the WHO goal of eliminating human rabies deaths by 2030. There is a need for a low-cost, readily reproducible method of estimating rabies burden and elimination capacity in endemic countries.

**Methods:**

Publicly available economic, environmental, political, social, public health, and One Health indicators were evaluated to identify variables with strong correlation to country-level rabies burden estimates. A novel index was developed to estimate infrastructural rabies elimination capacity and annual case-burden for dog-mediated rabies virus variant (DMRVV) endemic countries.

**Findings:**

Five country-level indicators with superior explanatory value represent the novel “STOP-R index:” (1) literacy rate, (2) infant mortality rate, (3) electricity access, (4) political stability, and (5) presence/severity of natural hazards. Based on the STOP-R index, 40,111 (95% CI 25,854–74,344) global human rabies deaths are estimated to occur in 2022 among DMRVV-endemic countries and are projected to decrease to 32,349 (95% CI 21,110–57,019) in 2030.

**Interpretation:**

The STOP-R index offers a unique means of addressing the data gap and monitoring progress towards eliminating dog-mediated human rabies deaths. Results presented here suggest that factors external to rabies programs influence the successes of rabies elimination, and it is now possible to identify countries exceeding or lagging in expected rabies control and elimination progress based on country infrastructure.

## Introduction

Rabies is a zoonotic disease that causes fatal encephalitis in humans if timely post-exposure prophylaxis (PEP) is not received (1–3). The global burden of the disease is momentous, affecting human health, animal health, and economic sectors (4). Though pre- and post-exposure prophylaxis are effective when utilized as recommended by the World Health Organization (WHO) (5), globally an estimated 59,000 human deaths occur each year, incurring an estimated 8.6 billion USD in economic losses (4). Rabies virus presents one of the highest risks for zoonotic disease spillover, and human deaths from rabies can be attributed to a complex combination of individual and systemic factors (6). These factors include: lack of local disease surveillance, underreporting or under-ascertainment of human exposure incidences, limited access to PEP, and low risk perception of disease severity and the necessity of PEP after a probable exposure event (1, 7, 8). Disparities in the accessibility of rabies PEP, often due to affordability and availability of vaccines, contribute to the global disease burden with greater impact in more impoverished, rural, or marginalized communities (4, 9, 10).

The dog-mediated rabies virus variant (DMRVV), transmitted predominantly through dog bites, is the most common source of human rabies infections (11). Robust public health measures have shown empirical success at controlling rabies in dog populations, including widespread vaccination of dogs and management of free-roaming dog populations (12–15). In 2018, the World Health Organization (WHO), the World Organization for Animal Health (WOAH, formerly known as OIE), the Food and Agriculture Organization of the United Nations (FAO), and the Global Alliance for Rabies Control (GARC) established a global initiative for the elimination of dog-mediated human rabies deaths by the year 2030 (“zero by 2030” or “ZB30”) (16). To monitor progress towards this goal and identify areas to maximize the impact of investments, it is critical to understand rabies burden and elimination capacity at country and regional levels.

The most commonly cited estimate of the global burden of rabies is based on country-reported data, reflective of a single time point (2013), modeled to derive burden estimates. This approach is difficult to update regularly due to cost, time, and data availability (4, 9). Global data repositories, such as the WOAH World Animal Health Information System (WAHIS) and WHO Global Health Observatory (GHO), receive voluntary reports of human and animal rabies cases (17, 18). However, rabies incidence data, when available, is typically acquired from passive surveillance systems, which capture rabies exposures and infections in individuals who are brought to the attention of healthcare providers or veterinarians; underrepresenting the true disease burden (19). Several studies have shown that passive public health systems under-detect human rabies cases by 10–100-fold and animal case by > 1,000-fold (20–22). Even if case detection were improved, reporting of cases to global repositories has low participation rates, thereby obscuring any data that may be available globally.

Thus, relying on one-time estimations, burdensome survey methods, and voluntary country reporting to surveillance repositories is not adequate to monitor progress towards the elimination of dog-mediated human rabies. Alternative, complementary burden estimation methods are necessary to overcome these data limitations (23–25). Monitoring the ZB30 goal requires effective monitoring of rabies trends at a periodicity that can inform global policy, at a reasonable cost. Monitoring trends in rabies epidemiology would help prioritise intervention methods, funding allocations, and improvements in healthcare services that are required to eliminate dog-mediated human rabies deaths by 2030. Therefore, there is a need for a low-cost, readily reproducible method of estimating rabies burden and elimination capacity in endemic countries.

In this study, a novel rabies index, the “STOP-R index,” was developed to estimate the rabies elimination potential and annual case-burden for all DMRVV-endemic countries. Given that accurate rabies surveillance data is routinely cited as a gap in nearly all endemic countries, our approach focused on assessing regularly collected and publicly available economic, environmental, political, social, public health, and One Health indicators. This type of data was evaluated to identify critical variables that have a strong correlation to country-level rabies burden estimates. This index makes it possible to evaluate the annual rabies burden and project future changes in disease burden at the country and regional levels. The five pillars of rabies elimination established by the WHO, WOAH, FAO, and GARC were used as a framework for this model, incorporating indicators that represent each pillar: Social, Technical, Organizational, Political, and Resource determinants (26). The specific aims of this study are to (1) determine if routinely available country-specific indicators can accurately predict dog-mediated human rabies burden, (2) create a novel index to estimate the current state of rabies elimination capacity, and (3) project trends in rabies elimination capacity and burden from 2022 to 2030.

## Methods

### Identification of countries eligible for analysis

Countries were eligible for inclusion in this analysis if they (1) had a recorded history of enzootic DMRVV (currently or historically enzootic), (2) have a national population greater than 100,000 people, and (3) had at least one empirically derived point estimate of rabies control capacity available in the selected global database.

Countries with human populations less than 100,000 were not considered for the analysis to prevent confounding results introduced by outliers such as small nations and isolated islands, as the approach to and success rate of canine rabies elimination was not thought to be similar to those required of larger endemic countries. Eligible countries were not weighted by human population or other country-specific parameters; each country eligible for this study was considered an equal observation when determining factors associated with rabies status.

### Identification of country-level measurements of dog-mediated rabies elimination capacity

A landscape review of global databases reflecting rabies elimination capacity or burden revealed four established resources: (1) WHO Global Health Observatory’s rabies assessment (27), (2) the Human Development Index’s correlation with dog rabies vaccine coverage and elimination (12), (3) 2015 Global Burden Study (4), and (4) outputs from country Stepwise Approach to Rabies Elimination (SARE) workshops. After qualitatively comparing country-level representativeness, relevance to the objectives of this study, and comparing subject matter expert opinions, country-specific parameters from the 2015 Global Burden Study were selected as the most appropriate values for the characterization of rabies control capacities (4). Three indicators from this study were selected as the most relevant for characterizing country capacities to control DMRVV: human rabies death rate (per 100,000 population), proportion of rabies exposures treated with PEP, and dog vaccination coverage (4). Each variable was transformed to reflect the relative value scaled from 0 to 100, with zero representing the most preferable value in the dataset (reflecting lower human rabies death rates, higher proportion of rabies exposures treated with PEP, and higher dog vaccination coverage) and 100 reflecting the least preferable value in the dataset (reflecting higher human rabies death rates, lower proportion of rabies exposures treated with PEP, and lower dog vaccination coverage). These three variables were combined through equal weighting summation and then re-scaled to reflect a value within the defined parameters. The resulting variable, referred to as the Global Burden Study-Rabies Susceptibility (GBS-RS) score, served as the dependent variable in this analysis. If a country eligible for analysis eliminated DMRVV prior to 2015, its GBS-RS score was set to zero. A lower GBS-RS score was assumed to reflect that the country is closer to rabies elimination while a higher GBS-RS score was assumed to reflect that the country is further from achieving rabies elimination.

### Identification and selection of infrastructure indicators

Indicators that influence the success of dog-mediated rabies elimination programs have been previously described as aligning with five categorical determinants (28): (1) Social, (2) Technical, (3) Organizational, (4) Political, and (5) Resource (Table 1).

**Table 1:** Description of the WOAH-defined five determinants of rabies elimination (STOP-R).

Determinant Category (WOAH-defined five pillars of rabies elimination)	Description^a^
Social Determinants	Rabies control involves a wide range of stakeholders, including the general public. The socio-cultural context influences rabies perception and dog-keeping practices. Understanding the context guides approaches to motivate behavioral change and plan feasible delivery of services.
Technical Determinants	Effective animal health and public health systems are required to eliminate dog-mediated human rabies. These systems must be strengthened and resourced appropriately, and gaps identified and filled.
Organizational Determinants	The One Health approach of close collaboration is applied. Leadership, public/private Partnerships, and coordination for rabies elimination activities are informed by the human health and animal health sectors and other stakeholders.
Political Determinants	Success is dependent on political climate and support for the elimination of dog-mediated human rabies. Political will results from recognition of rabies elimination as a national, regional, and global public good.
Resource Determinants	Rabies elimination activities frequently span several years and therefore require sustained, long-term support.

a Adapted from WHO; WOAH; FAO; GARC (28).

A landscape analysis was conducted to identify routinely collected, standardized, globally available infrastructural indicators aligned with the STOP-R determinants (Table 1). Bivariate linear regressions were conducted for the identified infrastructural indicators with GBS-RS score as the dependent variable. Results from bivariate regressions informed the use of each infrastructural indicator in multivariate analysis. Inclusion criteria for multivariate modelling of the infrastructural indicators included five considerations:

1. Infrastructural indicator must be available for the majority of eligible countries (*n* = 153).
2. *A priori* determination of plausibility that the indicator would impact dog-mediated rabies elimination capacity.
3. The bivariate association between the indicator and the GBS-RS; *R*^2^ values greater than 0.7 were considered highly correlated and therefore prioritized for model inclusion.
4. Indicator must not exhibit collinearity or high correlation with other indicators selected; when this occurred, subject matter experts determined inclusion of the indicator in the multivariate model.
5. Indicator must be aligned with the intent of at least one of the five determinants of rabies elimination from the ‘Global Framework for the Elimination of Dog-Mediated Human Rabies’ (STOP-R).

Missing values among indicators selected for analysis were replaced with the country’s United Nations-designated sub-regional average. Indicators for which a 2015 value was not available, the year closest was used for the analysis (2015).

### Development and assessment of a novel rabies index using STOP-R indicators

Infrastructural indicators were tested for multicollinearity by regressing the indicators against GBS-RS score. Collinearity was considered to exist if an indicator’s condition index was greater than 30 and two or more indicators showed variance decomposition proportions greater than 0.5. After removing indicators to address collinearity, a multivariate linear regression model was created to estimate the relationship between the selected infrastructural indicators and the country GBS-RS score. Several indicators that were not significantly associated with GBS-RS score in bivariate analyses were included in the fully adjusted multiple linear regression model based on *a priori* subject matter opinion (i.e., Natural Hazard Score, GARC Professionals). To achieve a parsimonious model, backwards elimination was conducted to reduce model parameters until reaching ‘best fit’ measured by adjusted R^2^ value, model value of p, and Akaike information criterion (AIC) (29). Criteria to consider during backwards elimination included the following:

1. Indicator significance value of p cutoff of < 0.05.
2. If removal of an indicator caused the model’s adjusted *R*^2^ (indicator of model’s fit) value to change > 1%, the indicator was kept in the model.
3. At least one independent indicator from each STOP-R category (from the ‘Global Framework for the Elimination of Dog-Mediated Human Rabies’) must remain the model, regardless of the data element’s significance (based on value of *p* and *R*^2^).
4. Final model must show a lower AIC value (indicator of model’s fit) than fully adjusted model.

Dummy variables were added into the final model to differentiate between the regional effects of Asia and Africa, the regions containing the highest human rabies death rates, compared to other regions. The final parsimonious model represents a novel rabies infrastructure index, the “STOP-R index” and enables estimation of annual country-specific rabies susceptibility scores. Lower STOP-R index values suggest that a country has infrastructure that is more conducive to supporting rabies control, whereas higher STOP-R index values suggest that a country has infrastructure that is less conducive to supporting rabies control efforts.

Country-specific STOP-R index values were regressed against their respective 2015 GBS-RS scores and the associated linear trendline and 95% prediction interval of the trend was determined. Country estimates that fell within the 95% prediction interval were considered to have developed rabies control capacity consistent with their expected infrastructural capabilities (classified as “aligned with infrastructural expectations”). Countries with higher GBS-RS scores compared to the STOP-R index values were considered to have lesser developed rabies control capacity than their infrastructural capabilities would suggest (classified as “lagging infrastructural expectations”). Countries with lower GBS-RS scores compared to the STOP-R index values were considered to have overcome barriers in infrastructural capabilities and developed rabies control capacity that exceeds expectations (classified as “exceeding infrastructural expectations”).

### Projection of expected rabies control progress and human deaths in 2030

To assess expected gaps and successes in the elimination of dog-mediated human rabies deaths, the five selected STOP-R index indicators were projected to the year 2030 on the country and sub-regional level (Supplementary Files 1–5). To do this, each indicator in the final STOP-R multivariate model underwent a country-level trend analysis in which ten preceding years of available data were analyzed for best-fitting trend functions, as evaluated by model *R*^2^ value. The trend functions were used to project the STOP-R model indicators’ values into the year 2030, and the STOP-R model equation was used to calculate country indices, using these values, for each year from 2022 to 2030.

For countries which are not DMRVV-free, country STOP-R index values were compared with human rabies death rates estimated by the Global Burden Study published in 2015 (4). Region-specific models were compared with country-aggregate models. The best-fitting functional region-specific equations, assessed by residuals standard error, *f*-statistic, and *R*^2^, were selected to represent the association between the STOP-R index and rate of human rabies deaths (per 100,000 population) (4). These functional equations were used to calculate country-specific human rabies deaths and death rates. Exponential/natural logarithm and polynomial functions showed high relatedness based on *R*^2^ value and were further evaluated to determine the best-fitting functions to calculate projected death rates (Supplementary Table 3). Residuals were calculated by subtracting the resulting STOP-R death rate by the Global Burden Study death rate for each country. Plots of the residuals by the STOP-R death rate were analyzed for each functional equation, as were the resulting global human rabies death estimates.

The final models were selected based on three factors: (1) a high *R*^2^ value when comparing the Global Burden Study human rabies death rate to the STOP-R index, (2) a high *R*^2^ value when comparing the residuals to the STOP-R death rate, and (3) a global rabies death estimate derived from the STOP-R index that was consistent with the current estimate used by the WHO to characterize global rabies burden, 59,000 (4). Country-specific dog-mediated human rabies deaths and accompanying 95% confidence intervals were calculated by applying STOP-R death rates by the projected country population during the years 2022 to 2030 (Supplementary Figure 1), while accounting for changing human populations during this time (30). It was assumed that improvements in the STOP-R index value would not immediately reflect gains in rabies control capacity. Therefore, death estimates were calculated based on a 5-year lag in STOP-R value (e.g., a 2015 STOP-R value would reflect the expected human rabies death rate in 2020) (12). Changes in country and sub-regional STOP-R index values were examined with consideration of the WHO’s 2030 elimination goal, and a STOP-R index “cutoff” was determined to mark the expectation of DMRVV-freedom for previously DMRVV-endemic countries (16). Bivariate and multivariate modelling was performed using SAS v9.4 (SAS Institute, Cary, NC, United States) and trend projections were calculated using Microsoft Excel (Microsoft Corporation, Redmond, Washington, DC, United States).

## Results

### Identification of eligible countries for analysis

Analysis was conducted on 153 countries that fit all inclusion criteria for analysis; 46 had eliminated DMRVV and 107 were endemic for DMRVV, as indicated in the 2015 Global Burden Study (4). Of the 18 UN-designated sub-regions represented by those 153 countries, 4 (Northern America, Northern Europe, Southern Europe, and Western Europe) had no countries with a presence of dog-mediated human rabies deaths as of 2015 (31).

### Identification of country-level measurements of dog-mediated rabies elimination capacity

**Figure 1:**
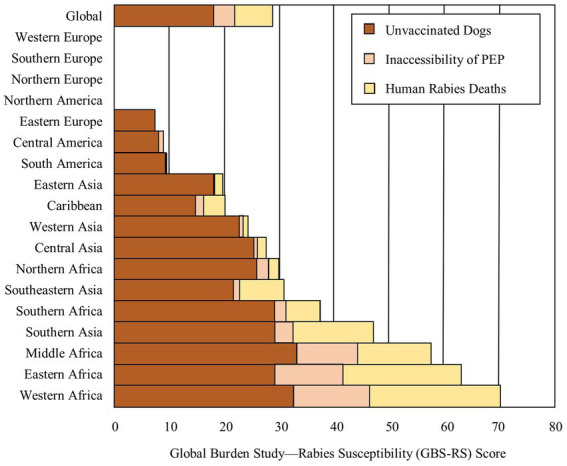
Distribution of average Global Burden Study-Rabies Susceptibility (GBS-RS) Score components among countries included in analysis by sub-regions.

With a possible range of 0–100, GBS-RS scores were calculated for 107 endemic countries and set as 0 for 46 countries that eliminated dog-mediated human rabies deaths. The average GBS-RS score was 28.9 with a range of 0–100 among all 153 countries included in the analysis. The average score was 41.4 with a range of 10.1–100 among only the 107 countries with a presence of DMRVV. Among the endemic countries, the proportion of unvaccinated dogs contributed to 63% of the GBS-RS score, human rabies death rate contributed to 24% of the GBS-RS score, and inaccessibility of rabies PEP contributed to 13% of the GBS-RS score (Figure 1). These distributions varied by sub-region.

### Identification of infrastructural indicators

**Figure 2:**
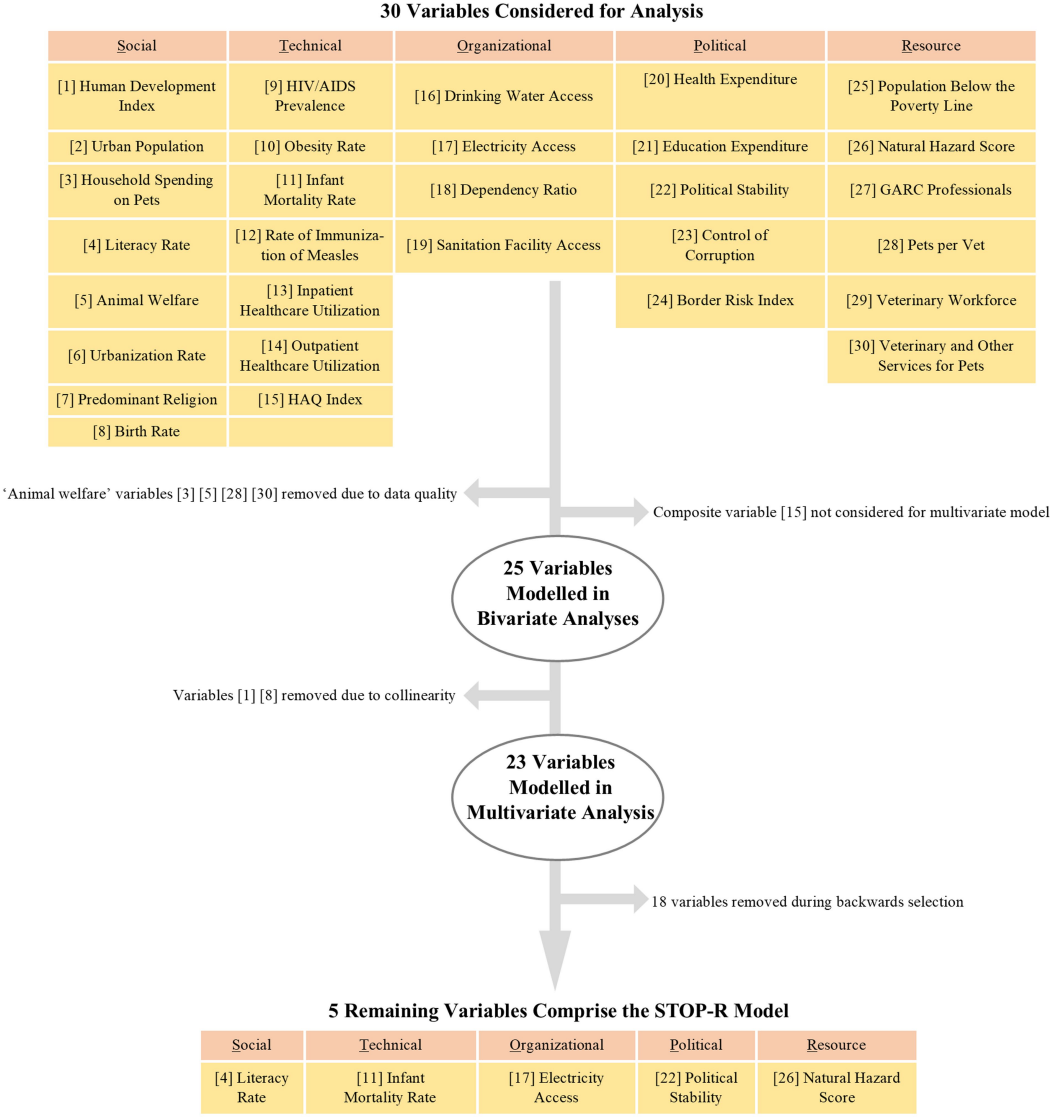
Independent variable selection process for bivariate and multivariate modelling. HAQ = Healthcare Access and Quality; GARC = Global Alliance for Rabies Control.

Thirty economic, environmental, political, social, public health, and One Health indicators fit the inclusion criteria for independent variables (Supplementary Table 1). Of the thirty indicators, ‘Animal Welfare,’ ‘Pets per Vet,’ ‘Veterinary and other Services for Pets,’ and ‘Household Spending on Pets’ were removed from the analysis due to poor data completeness (Figure 2). The ‘Healthcare Access and Quality (HAQ) index’ was removed because it is a composite indicator consisting of data elements that are individually represented in the model. Bivariate models ran on the remaining 25 indicators showed varying levels of associations between the indicators and GBS-RS score, with R^2^ values ranging from 0.31 to 0.85 (Supplementary Table 2). After testing for multicollinearity, the ‘Human Development Index’ and ‘Birth Rate’ indicators were removed.

### Development and assessment of a novel rabies index using STOP-R indicators

Multivariate linear regression modelling conducted on the remaining 23 indicators had an AIC of 744.5 and showed a good model fit with an adjusted *R*^2^ value of 0.86 (Table 2). The model resulting from backwards elimination, including a variable to differentiate between the regional effects of Asia and Africa, contained one indicator from each STOP-R category, had an AIC of 731.4, and showed a good model fit with an adjusted *R*^2^ value of 0.86. The indicator ‘Natural Hazard Score’ was the only indicator remaining in the final STOP-R model after backwards elimination that was not significantly associated with GBS-RS score, but was kept in the final model as the most significant representative within the ‘Resource’ category of determinants. The final model shows that GBS-RS score is positively associated with increases in infant mortality and frequency of natural hazards. Conversely, there is a negative association between GBS-RS score and decreases in literacy rate, political stability, and electricity access. In summary, capacity to eliminate rabies is expected to be lesser in countries with high rates of infant mortality, high frequency of natural hazards, lesser political stability, lower access to electricity, and lower literacy rates.

**Table 2:** Infrastructural indicator selection resulting in the STOP-R index model.

Indicator number^*^	Indicator	STOP-R category^**^	Fully adjusted model (Adj. *R*^2^ = 0.86)				STOP-R backwards elimination model (Adj. *R*^2^ = 0.86)			
			β Coefficient	95% CI		*p*-Value	β Coefficient	95% CI		*p*-Value
				Lower	Upper			Lower	Upper	
*Intercept*			57.3	16.2	98.4	0.007	75.4	55.3	95.5	<.0001
2	Urban population	S	0.0	−0.1	0.2	0.73				
4	Literacy rate		−0.2	−0.5	0.0	0.09	−0.3	−0.5	−0.2	<.0001
6	Urbanization rate		2.2	−0.3	4.7	0.09				
7	Predominant religion									
7	Christian		−11.9	−19.5	−4.4	0.002				
7	Muslim		−5.1	−13.0	2.9	0.21				
7	Other		*Referent category*							
9	HIV/AIDS prevalence	T	−0.3	−0.8	0.3	0.37				
10	Obesity rate		−0.2	−0.6	0.2	0.39				
11	Infant mortality rate		0.3	0.1	0.6	0.005	0.3	0.1	0.4	0.002
12	Rate of immunization of measles		0.2	0.0	0.4	0.01				
13	Inpatient healthcare utilization		21.1	−29.0	71.2	0.41				
14	Outpatient healthcare utilization		−0.4	−1.3	0.6	0.46				
16	Drinking water access	O	0.9	−0.5	2.2	0.21				
17	Electricity access		0.0	−0.2	0.3	0.70	−0.4	−0.5	−0.3	<.0001
18	Dependency ratio		−0.4	−0.5	−0.2	<.0001				
19	Sanitation facility access		−0.1	−0.4	0.1	0.31				
20	Health expenditure	P	−0.1	−0.3	0.1	0.22				
21	Education expenditure		0.2	−0.7	1.2	0.60				
22	Political stability		−3.5	−6.8	−0.2	0.04	−2.7	−5.1	−0.3	0.03
23	Control of corruption		−1.8	−5.4	1.7	0.31				
24	Border risk Index		0.1	−0.1	0.3	0.50				
25	Population below poverty line	R	5.0	−10.6	20.7	0.52				
26	Natural hazard score		−0.3	−1.8	1.1	0.64	0.3	−0.8	1.5	0.60
27	GARC professionals		0.0	0.0	0.0	0.30				
29	Veterinary workforce		0.0	0.0	0.0	0.18				
	Region									
	Africa						7.8	1.0	14.7	0.03
	Asia						14.6	10.0	19.2	<0.0001
	Other						*Referent category*			

* Indicator numbers are based on Figure 2.

** STOP-R category is based on the WOAH-defined five pillars of rabies elimination: S = social determinants, T = technical determinants, O = organizational determinants, P = political determinants, R = resource determinants.

STOP-R Index =

75.4 – (0.3*Literacy Rate) + (0.3*Infant Mortality Rate) – (0.4*Electricity Access) – (2.7*Political Stability) + (0.3*Natural Hazard Score)

+ 14.6 IF country in Asia

+ 7.8 IF country in Africa.

**Figure 3:**
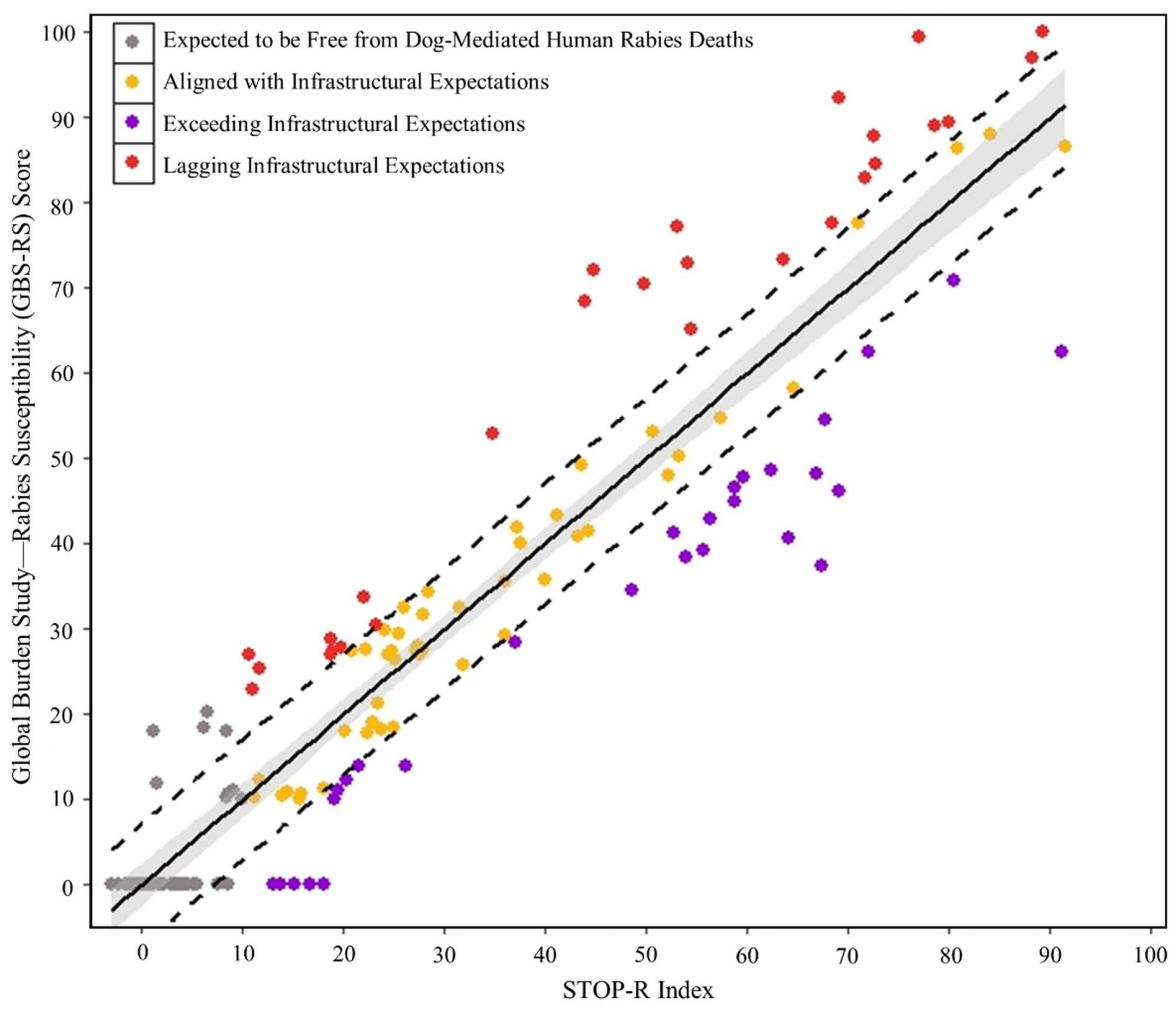
Categorical assessment of countries by infrastructure and rabies burden.

The final, fully adjusted model yielded the following coefficients to calculate country-specific STOP-R indices:

STOP-R index values among DMRVV-endemic and DMRVV-free locations ranged from −3.0 to 91.5 on the country level and from −0.5 to 69.2 on the sub-regional level for the year 2015 (Supplementary File 6). When the STOP-R index was compared to the GBS-RS score to determine how countries are performing in rabies control based on their infrastructural capabilities, excluding countries considered to be rabies-free, 28 countries (18%) were classified as ‘exceeding infrastructural expectations,’ 28 countries (18%) were classified as ‘lagging infrastructural expectations,’ and 47 countries (31%) were classified as ‘aligned with infrastructural expectations’ (Figure 3). A STOP-R index of 10 was assumed to represent a marker for the expectation of DMRVV-elimination upon examining trends in STOP-R index values for DMRVV-free countries in Figure 3, with 50 countries expected to be free from dog-mediated human rabies deaths based on their country infrastructure and rabies control capacity (Supplementary File 8).

### Projection of expected rabies control progress and human deaths in 2030

**Figure 4:**
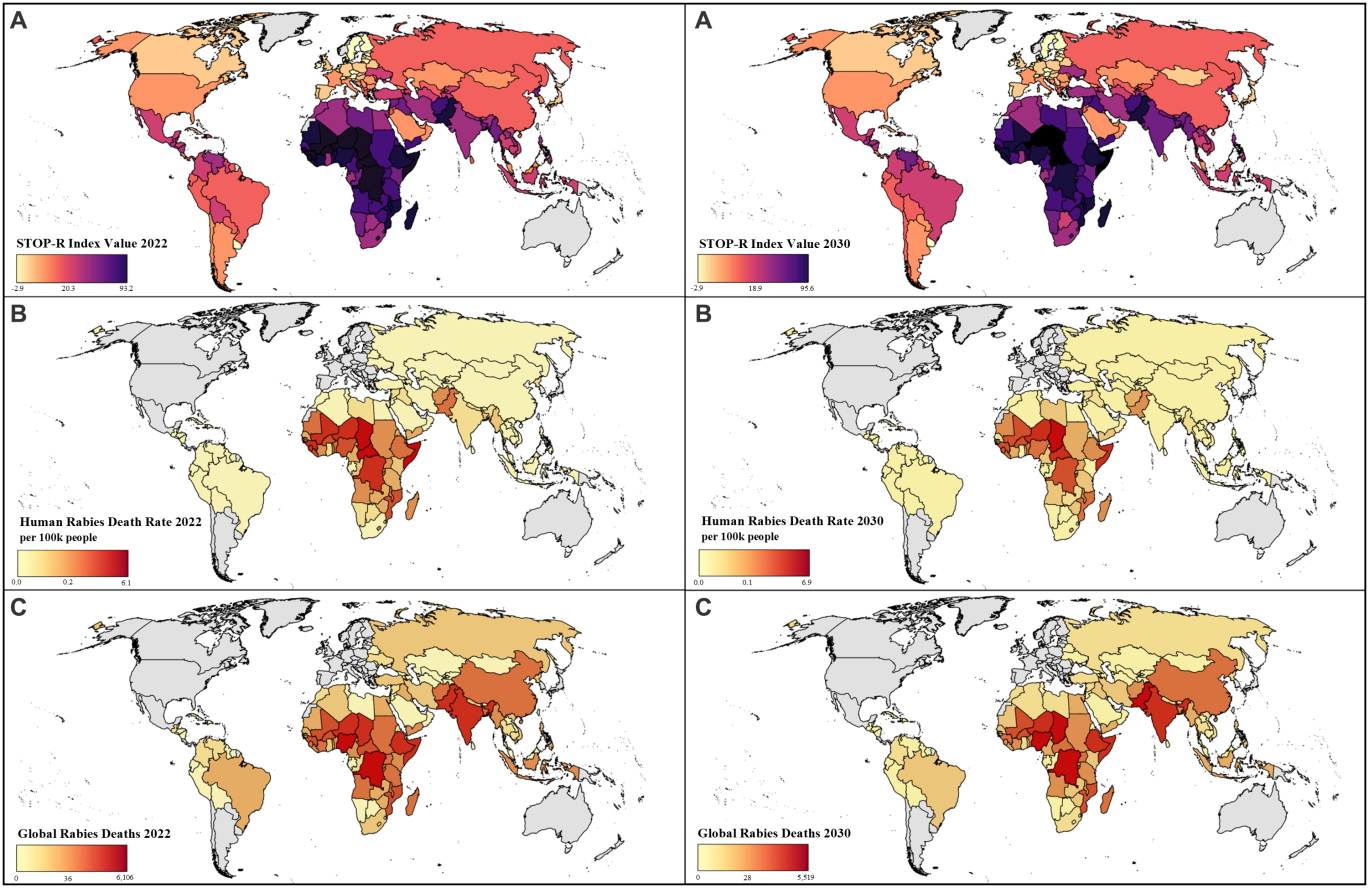
Projected human rabies deaths and STOP-R index values by country between 2022 and 2030:**(A)** Estimated STOP-R index values, **(B)** Estimated human rabies death rate per 100,000 people, **(C)** Global rabies deaths*. *Median values shown in legend. Countries in grey not included in analysis; death estimates were not calculated for countries without dog-mediated rabies virus variant in panels **B** and **C**; Mexico considered DMRVV-free as of 2019.

Projected STOP-R index values are predicted to increase for 12 countries from 2022 to 2030, indicating that country infrastructural capabilities are not expected to improve. STOP-R values are predicted to decrease for 136 countries, indicating that country infrastructural capabilities are expected to improve (Figure 4A; Supplementary File 6). On the sub-regional level, projected STOP-R index values decreased for all sub-regions containing endemic countries between 2022 and 2030, other than Northern America and Western Europe whose STOP-R index values are the same for those years (Supplementary File 7). Of sub-regions that are not DMRVV-free, the sub-region with the lowest projected STOP-R index in 2030 was Northern Europe and the sub-region with the highest was Western Africa.

**Figure 5:**
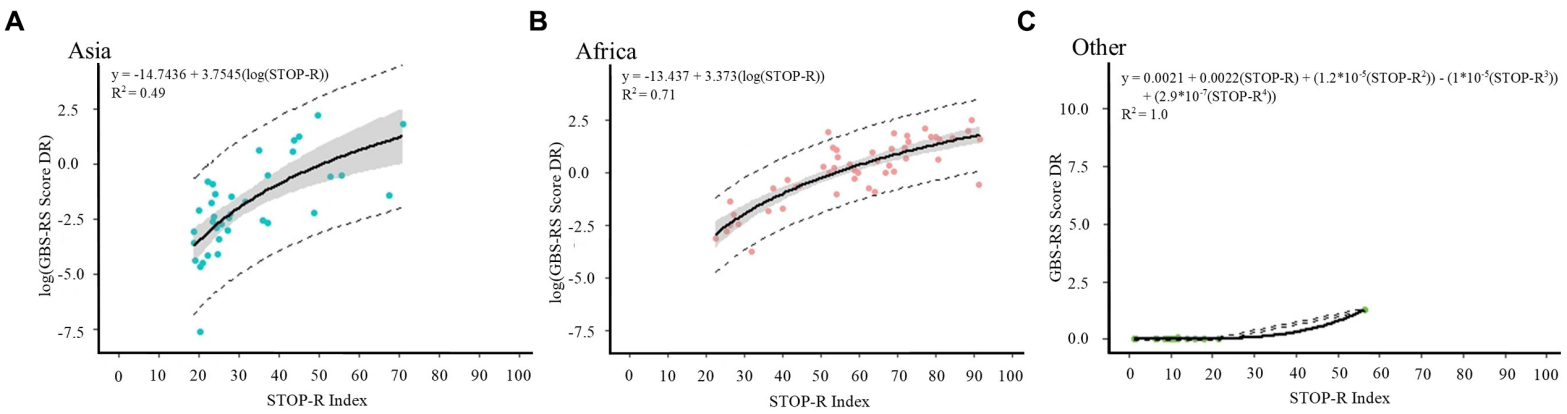
Models selected to achieve human rabies death rates and estimates for **(A)** Asia, **(B)** Africa, and **(C)** all other countries. GBS-RS Score DR=Global Burden Study-Rabies Susceptibility Score Death Rate.

Several models were compared to estimate human rabies deaths based on the STOP-R index model (Supplementary Table 3). Log–log functions were selected as the best fitting equations to calculate projected human rabies death rates for Asia and Africa, individually (Figure 5). A polynomial function of the fourth order was selected as the best fitting equation to calculate projected human rabies death rates for all other countries. These region-specific models showed better fit than aggregate, non-region-specific functions.

The country level projected human rabies death rate per 100,000 people in 2030 is estimated to range from 0.03 (95% CI 0.02–0.06) to 6.9 (95% CI 4.9–9.0; Figures 4B). Based on the STOP-R index, 40,111 (95% CI 25,854–74,344) human rabies deaths are estimated to occur in 2022 among DMRVV-endemic countries and are projected to decrease to 32,349 (95% CI 21,110–57,019) in 2030, assuming countries’ STOP-R indicator values follow historical and projected trends (Figure 4C; Supplementary File 9). Considering only sub-regions containing DMRVV-endemic countries, Southern Asia is estimated to experience the most human rabies deaths in 2022 with 13,931 (95% CI 7,969–38,362), followed by Western Africa with 9,933 (95% CI 6,934–11,885) and Eastern Africa with 7,492 (95% CI 5,332–8,987). The same ranking of sub-regions is seen in 2030 sub-regional human rabies death estimates (Supplementary File 10).

Seven countries which are currently classified as DMRVV-endemic are projected to have 0 human rabies deaths in 2030 and 34 additional countries are estimated to have less than 10 human rabies deaths in 2030. Based on projected 2030 STOP-R indices, 17endemic countries are expected to have infrastructural improvements consistent with that of rabies-free countries by 2030.

## Discussion

Rabies is a neglected disease, primarily due to poor detection probabilities stemming from limited field-level surveillance and diagnostic capabilities in most countries. As a result, there is limited ability to monitor and evaluate country, regional, and global progress towards the ZB30 goal. A global burden study was conducted in 2013 to estimate the burden of rabies and control capacity for nearly every endemic country (4). Studies such as these are critically important to developing effective policies for rabies control, but are expensive and logistically complicated to replicate on a routine basis. Additionally, endemic countries seldom report rabies surveillance the WHO Global Health Observatory, further obscuring the true burden and cost of dog-mediated rabies; human rabies death estimates were only reported by 93 of 194 countries in 2017 (18). Recognizing that widespread improvements in the infrastructure for adequate surveillance and diagnostic capabilities is unlikely to be realized in the near future, indirect burden estimations are an unfortunate necessity to obtain yearly estimates. Indirect estimation methods using composite indices of better-understood variables are not uncommon; many composite variables are routinely used to monitor political, economic, and health outcomes, including the Human Development Index, Gross Domestic Product, and Environmental Sustainability Index. This study has attempted to establish one such indirect estimation for monitoring rabies burden over time, and the results reveal a promising method for systematic, annual rabies burden re-estimation. Until global rabies surveillance and data sharing systems are improved, these indirect methods provide insight into progress toward the WHO ZB30 goal.

There have been numerous attempts to estimate dog-mediated human rabies deaths over the past several decades. Due to underreporting of human rabies deaths, all of these methods have relied on mathematical models to derive estimates. Additionally, a 2020 global systematic review of rabies spatial epidemiology data concluded that the literature currently lacks appropriate methodology and data to provide an evidence-based approach towards dog-mediated rabies elimination efforts (32). Various modeling methods have resulted in a wide variation in annual human death estimates, ranging from 13,000 to 60,000 (33–35). The methods used here also rely on mathematical modelling and provide estimates consistent with previously published point estimates.

One publication, a global rabies burden analysis published in 2015, is consistently referenced for global rabies statistics throughout literature, the media, and scientific discussions (4). The authors estimated that globally 59,000 (95% CI 25,000–159,200) human deaths from rabies occurred in 2015, which is consistently referred to as an annual, instead of one-time, estimate in a considerable number of publications (4). Referencing this figure as a current estimate does not enable the rabies community to showcase progress towards the elimination of dog-mediated human rabies deaths since the study’s publication; examples of rabies control improvements are continuously reported (36–40). Assuming trends in the STOP-R index values continue as expected and no major global or national disruptions to these extrinsic and intrinsic factors occur, there will be an estimated 32,349 (95% CI 21,110–57,019) human rabies deaths in 2030. Results presented here suggest that the goal of elimination of dog-mediated human rabies deaths will not be reached without a significant change in multi-sectoral interest and support. However, these results should encourage communities, national governments, and international agencies to lend focused support for rabies control to address these infrastructural gaps and follow the lead of countries that have shown it to be possible to eliminate rabies or overcome infrastructural challenges.

Five indicators representing the five determinants of the rabies elimination framework showed superior explanatory value and were chosen to represent the STOP-R index: (1) literacy rate, (2) infant mortality rate, (3) electricity access, (4) political stability, and (5) presence and severity of natural hazards.

1. Literacy rate, which is associated with increased country capacity to eliminate rabies, is an indicator of country educational development. Those in endemic countries are likely to be more empowered to recognize rabies exposures, seek PEP, and vaccinate their dogs if they understand why these public health actions are important (41).
2. Infant mortality, which is associated with decreased country capacity to eliminate rabies, is a standard indicator of poverty and a technical determinant reflecting the state of health systems (42). Countries with relatively high rates of infant mortality are expected to have increased competition for public health resources, which may negatively impact the success of a rabies program.
3. Lower rates of access to electricity are associated with decreased country capacity to eliminate rabies. Without access to electricity, the cold chain required for human and dog vaccine storage cannot be maintained, among other difficulties that would complicate dog vaccination campaigns, other rabies control activities, and other One Health activities. Electrical access likely reflects the broader infrastructure of a country, and lower infrastructural development would make all aspects of rabies prevention and control more difficult.
4. Political stability is associated with increased country capacity to eliminate rabies. Political instability often causes economic instability, which can cause adverse ripple-effects throughout a country or region and set back political initiatives like disease control programs (43).
5. Higher frequency of natural hazards is associated with decreased country capacity to eliminate rabies. These events can hamper rabies prevention and control activities in a myriad of ways; for example, by resulting in the displacement of dog populations and impeding dog vaccination programs (44).

Extrinsic threats, which likely cannot be controlled to a meaningful degree by a national rabies control program, include natural hazards, global political disruptions, and significant economic disasters. The most basic concern about population disrupting events, such as global pandemics or natural disasters, is the diversion of efforts and resources that might otherwise be used for rabies control activities—as was experienced during the COVID-19 pandemic (45–47). The incidences of natural catastrophic events are predicted to increase as global temperatures continue to rise, with most significant impacts affecting many of the most heavily rabies-affected countries. The resulting disruption of health programs and redistribution of public resources will likely be a source of competition for rabies control efforts in the coming years and decades (48). Depending on geographic location, some countries are inherently at greater risk of impact from these extrinsic factors. Annual re-assessment of the STOP-R indicators and re-estimation of rabies burden will be even more important as the frequency of these global emergencies increases.

Intrinsic threats, which are largely under the control or influence of national governments, consist of deficient investments in key societal markers: literacy, infant mortality rate, and political stability. Intrinsic threats require that governments prioritize resources, appropriately govern, and show commitment to sustainable improvements for progress to be achieved. Since advancements in these intrinsic indicators would likely result in downstream benefits to rabies control programs, an all-systems approach to advocacy in which zoonotic disease programs are also considered in societal investments may offer a means of mutual cross-program synergy (49).

The STOP-R index is a unique tool for assessing rabies burden because it reflects holistic country infrastructure development relative to rabies control capacity. This type of analytical process allows countries to be identified that might be suitable for prioritizing domestic and international investment in rabies control activities. For example, a country with a relatively low STOP-R index value compared to their expected rabies burden (“exceeding infrastructural expectations”) may reflect a good investment for rabies control, as they have overcome infrastructural barriers to develop rabies control capacity. However, these methods cannot predict several important determinants of a successful rabies program, including political will, external support, and community engagement, amongst others. These factors may play a significant role in countries with the infrastructure to support rabies control activities but have not yet done so.

It is unlikely that improvements in the core infrastructural indicators that make up the STOP-R index would immediately result in changes in rabies control capacity and associated death rates, and it is also unlikely that there is a standard time delay across all countries. However, this analysis assumes a 5-year delay from when a STOP-R index value changes until this change affects the rabies program. Additionally, it is reasonable to assume that worsening STOP-R values would more immediately impact rabies control programs compared to improvements in STOP-R values, as was evident during the COVID-19 pandemic when several countries reported spikes in rabies cases shortly after rabies control activities were halted (50, 51). The COVID-19 pandemic temporarily disrupted dog vaccination programs in many countries; lapsed vaccination campaigns can lead to rapid declines in vaccine herd immunity and rapidly derail vaccination program successes that may take years to develop (48). Future applications of this model to predict changes in rabies burden may wish to consider different delay periods, particularly in situations where STOP-R index values are expected to worsen.

While STOP-R is a useful tool for better understanding country, regional, and global rabies control capacity and burden, it was created due to the lack of global data on rabies incidence. Therefore, in countries where evaluations of current rabies capacity and incidence are available, validated estimates should be used instead of the estimation method described here. Countries with better infrastructure that are lacking in rabies control warrant further investigation to determine specific, underlying reasons for non-participation in rabies control activities on a case-by-case basis. This analysis is based on 2015 data, and it is possible that countries have since achieved elimination of dog-mediated human rabies deaths, or that countries made significant changes in the Global Burden Study indicators used here; these changes would not be reflected in this model, and could lead to erroneous conclusions. Finally, the development of the STOP-R does not address the pivotal issue in rabies elimination: poor data quality. Countries should consider implementing programs that improve field-level data collection and participate in global data sharing platforms like the WHO Global Health Observatory.

This proposed STOP-R index for estimating rabies susceptibility is not preferred; it is an unfortunate necessity given the weak state of rabies surveillance in most endemic countries. The STOP-R model appears to be a robust means of estimating changes in rabies burden and offers a unique means of addressing the data gap and monitoring progress towards eliminating dog-mediated human rabies deaths. Results from this analysis suggest that factors external to rabies programs likely influence the successes of rabies elimination, and it is now possible to identify countries that are exceeding or lagging in expected rabies control and elimination progress based on country infrastructure. According to the results presented here, the ZB30 goal is unlikely to be met without a drastic increase in support for rabies control activities and without fundamental infrastructural improvements in many endemic countries. However, the rabies community is continuously changing with attention and expertise from groups that aim to accelerate progress towards the ZB30 goal. For example, The United Against Rabies Forum was recently developed to provide the impetus needed to promote collaboration among technical experts, increase political engagement, and mobilize resources (52). Committed and continued injections of energy towards this global goal may ultimately be the reason for future global elimination success.

## Data availability statement

The original contributions presented in the study are included in the article/Supplementary material, further inquiries can be directed to the corresponding author.

## Author contributions

SB: literature search, figures, study design, data collection, data analysis, data interpretation, original writing, and writing—review and editing. JM: literature search, data collection, data analysis, data interpretation, original writing, and writing—review and editing. EU: study design, data interpretation, and writing—review and editing. RW: figures, study design, data collection, data analysis, data interpretation, original writing, and writing—review and editing. All authors contributed to the article and approved the submitted version.

## Conflict of interest

The authors declare that the research was conducted in the absence of any commercial or financial relationships that could be construed as a potential conflict of interest.

## Publisher’s note

All claims expressed in this article are solely those of the authors and do not necessarily represent those of their affiliated organizations, or those of the publisher, the editors and the reviewers. Any product that may be evaluated in this article, or claim that may be made by its manufacturer, is not guaranteed or endorsed by the publisher.

## Author disclaimer

The conclusions, findings, and opinions expressed by authors contributing to this journal do not necessarily reflect the official position of the U.S. Department of Health and Human Services, the Public Health Service, the Centers for Disease Control and Prevention, or the authors’ affiliated institutions.

## References

[1] Fooks AR Banyard AC Horton DL Johnson N McElhinney LM Jackson AC. Current status of rabies and prospects for elimination. Lancet. (2014) 384:1389–99. doi: 10.1016/S0140-6736(13)62707-5, PMID: 24828901 PMC7159301

[2] Warrell MJ Warrell DA. Rabies: the clinical features, management and prevention of the classic zoonosis. Clin Med (Lond). (2015) 15:78–81. doi: 10.7861/clinmedicine.14-6-78, PMID: 25650205 PMC4954532

[3] Johnson N Vos A Freuling C Tordo N Fooks A Müller T. Human rabies due to lyssavirus infection of bat origin. Vet Microbiol. (2010) 142:151–9. doi: 10.1016/j.vetmic.2010.02.001, PMID: 20188498

[4] Hampson K Coudeville L Lembo T Sambo M Kieffer A Attlan M. Estimating the global burden of endemic canine rabies. PLoS Negl Trop Dis. (2015) 9:e0003709. doi: 10.1371/journal.pntd.0003709, PMID: 25881058 PMC4400070

[5] Pattanaik A Mani RS. WHOʼs new rabies recommendations. Curr Opin Infect Dis. (2019) 32:401–6. doi: 10.1097/QCO.0000000000000578, PMID: 31305491

[6] Grange ZL Goldstein T Johnson CK Anthony S Gilardi K Daszak P. University of Edinburgh Epigroup members those who wish to remain anonymous. Ranking the risk of animal-to-human spillover for newly discovered viruses. Proc Natl Acad Sci U S A. (2021) 118:e2002324118. doi: 10.1073/pnas.2002324118, PMID: 33822740 PMC8053939

[7] Nel LH Taylor LH Balaram D Doyle KAS. Global partnerships are critical to advance the control of neglected zoonotic diseases: the case of the global alliance for rabies control. Acta Trop. (2017) 165:274–9. doi: 10.1016/j.actatropica.2015.10.014, PMID: 26519885

[8] Taylor LH Knofp L. Surveillance of human rabies by national authorities–a global survey. Zoonoses Public Health. (2015) 62:543–52. doi: 10.1111/zph.12183 25683444

[9] Knobel DL Cleaveland S Coleman PG Fevre EM Meltzer MI Miranda ME. Re-evaluating the burden of rabies in Africa and Asia. Bull World Health Organ. (2005) 83:360–8. 15976877 PMC2626230

[10] Rupprecht CE Salahuddin N. Current status of human rabies prevention: remaining barriers to global biologics accessibility and disease elimination. Expert Rev Vaccines. (2019) 18:629–40. doi: 10.1080/14760584.2019.1627205, PMID: 31159618

[11] Hampson K Dushoff J Cleaveland S Haydon DT Kaare M Packer C. Transmission dynamics and prospects for the elimination of canine rabies. PLoS Biol. (2009) 7:e1000053:e53. doi: 10.1371/journal.pbio.1000053, PMID: 19278295 PMC2653555

[12] Wallace RM Undurraga EA Blanton JD Cleaton J Franka R. Elimination of dog-mediated human Rabies deaths by 2030: needs assessment and alternatives for Progress based on dog vaccination. Front Vet Sci. (2017) 4:9. doi: 10.3389/fvets.2017.00009 28239608 PMC5300989

[13] Taylor LH Wallace RM Balaram D Lindenmayer JM Eckery DC Mutonono-Watkiss B. The role of dog population Management in Rabies Elimination—A Review of current approaches and future opportunities. Front Vet Sci. (2017) 4:109. doi: 10.3389/fvets.2017.00109, PMID: 28740850 PMC5502273

[14] González-Roldán JF Undurraga EA Meltzer MI Atkins C Vargas-Pino F Gutiérrez-Cedillo V. Cost-effectiveness of the national dog rabies prevention and control program in Mexico, 1990-2015. PLoS Negl Trop Dis. (2021) 15:e0009130. doi: 10.1371/journal.pntd.0009130, PMID: 33661891 PMC7963054

[15] Vigilato MAN Clavijo A Knobl T Silva HMT Cosivi O Schneider MC. Progress towards eliminating canine rabies: policies and perspectives from Latin America and the Caribbean. Philos Trans R Soc Lond B Biol Sci. (2013) 368:20120143. doi: 10.1098/rstb.2012.0143, PMID: 23798691 PMC3720041

[16] World Health Organization. Zero by 30: The global strategic plan to prevent human deaths from dog-transmitted rabies by 2030—Executive summary. (2018). Available at: http://www.oie.int/fileadmin/Home/eng/Media_Center/docs/pdf/Rabies_portal/EN_executiveSummary.pdf

[17] WOAH (OIE). World Animal Health Information System (2020). Available at: https://wahis.oie.int/

[18] WHO. Global Health Observatory (2023). Available at: https://www.who.int/data/gho

[19] Gibbons CL Mangen M-JJ Plass D Havelaar AH Brooke RJ Kramarz P. Measuring underreporting and under-ascertainment in infectious disease datasets: a comparison of methods. BMC Public Health. (2014) 14:1–17. doi: 10.1186/1471-2458-14-147 24517715 PMC4015559

[20] Taylor LH Hampson K Fahrion A Abela-Ridder B Nel LH. Difficulties in estimating the human burden of canine rabies. Acta Trop. (2017) 165:133–40. doi: 10.1016/j.actatropica.2015.12.007, PMID: 26721555 PMC5178864

[21] Wallace RM Blanton J. Chapter 4–Epidemiology. In: Fooks AR Jackson Rabies AC, editors. 4th ed: Academic Press (2020). 103–42.

[22] Townsend SE Lembo T Cleaveland S Meslin FX Miranda ME Putra AAG. Surveillance guidelines for disease elimination: A case study of canine rabies. Comp Immunol Microbiol Infect Dis. (2013) 36:249–61. doi: 10.1016/j.cimid.2012.10.008, PMID: 23260376 PMC3693035

[23] Cleavleland S Lankester F Townsend S Lembo T Hampson K. Rabies control and elimination: a test case for one health. Vet Rec. (2014) 175:188–93. doi: 10.1136/vr.g4996, PMID: 25172649 PMC7612423

[24] Lavan RP King AI Sutton DJ Tunceli K. Rationale and support for a one health program for canine vaccination as the most cost-effective means of controlling zoonotic rabies in endemic settings. Vaccine. (2017) 35:1668–74. doi: 10.1016/j.vaccine.2017.02.014, PMID: 28216188

[25] Nel LH. Discrepancies in data reporting for rabies. Africa Emerg Infect Dis. (2013) 19:529–33. doi: 10.3201/eid1904.120185 23628197 PMC3647406

[26] WOAH (OIE). Global strategic framework for the elimination of dog-mediated human rabies. (2016). Available at: https://www.oie.int/en/global-strategic-framework-for-the-elimination-of-dog-mediated-human-rabies/

[27] WHO Global Health Observatory. Reported number of human rabies deaths. (2022) Available at: https://www.who.int/data/gho/data/indicators/indicator-details/GHO/reported-number-of-human-rabies-deaths

[28] WHO; WOAH; FAO; GARC. Global framework for the elimination of dog-mediated human rabies. Available at: https://www.who.int/rabies/control/Poster_Global_framework_for_the_elimination_of_dog-mediated_human_rabies.pdf

[29] Kleinbaum D. G. Kupper L. L. Nizam A. Rosenberg E. S. (2014). Applied regression analysis and other multivariable methods (5th). Bosto, MA: Cengage Learning, p447–449.

[30] The World Bank. World development indicators: Population growth (annual %). (2021). Available at: https://data.worldbank.org/indicator/SP.POP.GROW

[31] United Nations Statistical Division. Methodology: Standard country or area codes for statistical use (M49). (2022). Available at: https://unstats.un.org/unsd/methodology/m49/

[32] Mshelbwala P Weese S Mamun A Magalhaes R. Global spatial epidemiology of rabies: systematic review and critical appraisal of methods. Int J Infect Dis. (2020) 101:321. doi: 10.1016/j.ijid.2020.09.839

[33] Meslin FX Briggs DJ. Eliminating canine rabies, the principal source of human infection: what will it take? Antivir Res. (2013) 98:291–6. doi: 10.1016/j.antiviral.2013.03.011, PMID: 23523768

[34] James SL Abate D Abate KH Abay SM Abbafati C Abbasi N. Global, regional, and national incidence, prevalence, and years lived with disability for 354 diseases and injuries for 195 countries and territories, 1990-2017: a systematic analysis for the global burden of disease study 2017. Lancet. (2018) 392:1789–858. doi: 10.1016/S0140-6736(18)32279-7, PMID: 30496104 PMC6227754

[35] Vos T Abajobir AA Abate KH Abbafati C Abbas KM Abd-Allah F. Global, regional, and national incidence, prevalence, and years lived with disability for 328 diseases and injuries for 195 countries, 1990-2016: a systematic analysis for the global burden of disease study 2016. Lancet. (2017) 390:1211–59. doi: 10.1016/S0140-6736(17)32154-2, PMID: 28919117 PMC5605509

[36] Ceballos NA Rodríguez PP Setién ÁA. The new face of human Rabies in Mexico, What's next after eradicating Rabies in dogs. Vector Borne Zoonotic Dis. (2022) 22:69–75. doi: 10.1089/vbz.2021.0051, PMID: 35175137

[37] Gibson AD Wallace RM Rahman A Bharti OK Isloor S Lohr F. Reviewing solutions of scale for canine Rabies elimination in India. Trop Med Inf Dis. (2020) 5:47. doi: 10.3390/tropicalmed5010047, PMID: 32210019 PMC7157614

[38] Adrien J Georges Y Augustin PD Monroe B Gibson AD Fenelon N. Notes from the field: a multipartner response to prevent a binational Rabies outbreak—Anse-à-Pitre, Haiti, 2019. MMWR Morb Mortal Wkly Rep. (2019) 68:707–9. doi: 10.15585/mmwr.mm6832a6, PMID: 31415489 PMC6818699

[39] Yin W Fu ZF Gao GF. Progress and prospects of dog-mediated Rabies elimination in China. China CDC Weekly. (2021) 3:831–4. doi: 10.46234/ccdcw2021.205, PMID: 34595002 PMC8477050

[40] Thai Rabies Net. (2023). Available at: http://thairabies.net

[41] Dewalt DA Berkman ND Sheridan S Lohr KN Pignone MP. Literacy and health outcomes: a systematic review of the literature. J Gen Intern Med (2004);19:1228–1239. doi: 10.1111/j.1525-1497.2004.40153.x 15610334 PMC1492599

[42] Reidpath DD. Allotey PInfant mortality rate as an indicator of population healthJournal of. Epidemiol Commun Health. (2003) 57:344–6. doi: 10.1136/jech.57.5.344, PMID: 12700217 PMC1732453

[43] Klomp J de Haan J. Is the political system really related to health? Soc Sci Med. (2009) 69:36–46. doi: 10.1016/j.socscimed.2009.03.033 19427088

[44] Rabies in Manmade or Natural Disasters. Centers for Dsiease control and prevention (2011). Available at: https://www.cdc.gov/rabies/specific_groups/veterinarians/disasters.html

[45] Kunkel A Jeon S Joseph HC Dilius P Crowdis K Meltzer MI. The urgency of resuming disrupted dog rabies vaccination campaigns: a modeling and cost-effectiveness analysis. Sci Rep. (2021) 11:12476. doi: 10.1038/s41598-021-92067-5, PMID: 34127783 PMC8203735

[46] Raynor B Díaz EW Shinnick J Zegarra E Monroy Y Mena C. The impact of the COVID-19 pandemic on rabies reemergence in Latin America: the case of Arequipa. Peru PLOS Neglected Trop Dis. (2021) 15:e0009414. doi: 10.1371/journal.pntd.0009414, PMID: 34019548 PMC8174740

[47] Ma X Bonaparte S Toro M Orciari LA Gigante CM Kirby JD. Rabies surveillance in the United States during 2020. J Am Vet Med Assoc. (2022) 260:1–9. doi: 10.2460/javma.22.03.0112, PMID: 35522584

[48] Diaz JH. The influence of global warming on natural disasters and their public health outcomes. Am J Disaster Med. (2007) 2:33–42. doi: 10.5055/ajdm.2007.0007 18268873

[49] Nadal D Beeching S Cleaveland S Cronin K Hampson K Steenson R. Rabies and the pandemic: lessons for one health. Trans R Soc Trop Med Hyg. (2022) 116:197–200. doi: 10.1093/trstmh/trab123, PMID: 34392375 PMC8890778

[50] The Times of India. Rabies cases in Maharashtra doubled during first year of pandemic: RTI data. (2022). Available at: https://timesofindia.indiatimes.com/city/pune/rabies-cases-in-maharashtra-doubled-during-first-year-of-pandemic-rti-data/articleshow/90529374.cms

[51] Outbreak News Today (2022). Philippines: Rabies deaths up 157% in northern Mindanao, Most in Bukidnon. Philippines: Rabies deaths up 157% in Northern Mindanao, Most in Bukidnon–Outbreak News Today

[52] Tidman R Thumbi SM Wallace R de Balogh K Iwar V Dieuzy-Labaye I. United against Rabies forum: the one health concept at work. Front Public Health. (2022) 10:854419. doi: 10.3389/fpubh.2022.854419, PMID: 35493394 PMC9043483

